# Development of a Sex-Specific Prevalent Hypertension Discrimination Model in Korean Adults Using Genetic Risk Scores and Clinical Biomarkers: A Cross-Sectional Study

**DOI:** 10.3390/cimb48030271

**Published:** 2026-03-03

**Authors:** Jua Park, Ximei Huang, Minjoo Kim

**Affiliations:** Department of Food and Nutrition, College of Life Science and Nano Technology, Hannam University, Daejeon 34054, Republic of Korea

**Keywords:** hypertension, genetic risk score, prediction model, single-nucleotide polymorphism, pulse wave velocity

## Abstract

Genetic and metabolic factors contribute to hypertension, yet integrated sex-specific models remain limited. In this cross-sectional study, we developed sex-specific models to discriminate prevalent hypertension discrimination by integrating genetic risk scores (GRSs) with metabolic and vascular biomarkers. From 2075 Korean adults, the final models were evaluated using model-specific complete-case datasets (total *n* = 775; males *n* = 382; females *n* = 397). Blood pressure-related single-nucleotide polymorphisms (SNPs) were screened using genome-wide association analyses (*p* < 1 × 10^−5^), and selected variants were used to construct weighted GRSs. Models integrating GRSs with body mass index (BMI), brachial–ankle pulse wave velocity (ba-PWV), and urinary 8-epi-prostaglandin F_2α_ (8-epi-PGF_2α_) were evaluated by multivariable logistic regression and receiver operating characteristic analysis, with 1000-bootstrap internal validation. Three SNPs formed the total-sample GRS (rs13175330, rs117559502, rs62099117; adjusted odds ratio [OR] = 3.20) and three formed the female GRS (rs13175330, rs6001482, rs62099117; adjusted OR = 2.80); no stable male-specific GRS met prespecified criteria. The final discrimination models achieved an area under the curve of 0.833 in the total sample and 0.913 in females (BMI + ba-PWV + 8-epi-PGF_2α_ + GRS), and 0.758 in males (BMI + ba-PWV + 8-epi-PGF_2α_). These findings support sex-aware hypertension risk characterization and warrant external and prospective validation.

## 1. Introduction

Hypertension affects over 1.2 billion people worldwide and remains the leading modifiable risk factor for cardiovascular morbidity and mortality [1]. Its prevalence continues to rise due to demographic and lifestyle changes—including population aging, increasing obesity, sedentary behavior, and poor dietary patterns—exacerbating cardiovascular risks and premature death [2]. Epidemiological studies consistently demonstrate sex disparities in hypertension prevalence, with males exhibiting higher rates than females across diverse populations [3]. In South Korea, similar trends are observed: the 2024 Korea Hypertension Fact Sheet reports an increase in adult hypertension prevalence from 26.7% in 2012 to 30.1% in 2022, with higher rates in men (33.4%) than in women (26.8%) [4]. These sex-specific disparities have significant clinical implications for risk prediction and management.

While hormonal and behavioral factors are well-recognized contributors to these disparities, emerging evidence highlights the importance of genetic mechanisms [5]. Large-scale genome-wide association studies (GWASs) have collectively identified over 3500 blood pressure (BP)-associated single-nucleotide polymorphisms (SNPs), confirming the polygenic nature of hypertension [6]. Notably, sex-stratified GWAS have revealed that females show stronger associations between BP-related SNPs and hypertension risk [7], emphasizing the need for sex-specific genetic risk models. Advances in precision medicine enable deeper investigation of these genetic determinants. While individual SNPs have limited predictive capacity, genetic risk scores (GRS) that integrate multiple SNPs’ effects offer a more comprehensive assessment of genetic susceptibility [8]. Such approaches facilitate the exploration of the molecular mechanisms underlying individual risk profiles [9].

Despite these advances, most existing hypertension prediction models continue to rely on conventional clinical and biochemical indicators due to their accessibility and practicality [10]. These metabolic biomarkers—including lipid profiles, glycemic indices, inflammatory markers, and oxidative stress indicators—provide valuable insights into the early vascular and metabolic dysfunction underlying the pathogenesis of hypertension [11].

Given the complementary roles of genetic and metabolic markers in hypertension development, integrating both into a unified risk discrimination model represents a promising strategy for precision prevention. Accordingly, the present study aims to develop novel sex-specific models to discriminate prevalent hypertension by combining GRS and metabolic biomarkers in a Korean population, with the ultimate goal of advancing individualized risk stratification and enabling population-targeted prevention strategies.

## 2. Materials and Methods

### 2.1. Study Population

This study is a secondary analysis of the National Health Insurance Corporation Ilsan Hospital health screening cohort in Goyang, Korea, which has been described previously [12]. Health screening data used for the present analysis were collected between January 2010 and March 2015. The current work is a cross-sectional model-development study using data from the baseline (first eligible) health screening visit within this period, rather than a longitudinal follow-up analysis. The analytic dataset was extracted and de-identified prior to analysis, and statistical analyses were conducted between January 2025 and January 2026. The study protocol was approved by the Institutional Review Board of Hannam University (2023-04-08-0405) and conducted under the principles of the Declaration of Helsinki. Participants provided written informed consent at cohort enrollment for the research use of their data.

Participants were recruited based on their health check-up data, and those who consented to participate underwent additional health assessments by the Department of Family Medicine. Eligible participants were selected according to prespecified criteria to define an analytic cohort suitable for model development and to reduce major sources of treatment-related distortion and improve internal validity in model development. Hypertension was classified based on the guidelines from the Korean Society of Hypertension, defined as systolic BP ≥ 140 mmHg or diastolic BP ≥ 90 mmHg, and/or current use of antihypertensive medication [13], ascertained by a standardized questionnaire. Individuals receiving antihypertensive treatment were classified as having hypertension regardless of their measured BP at the examination.

Individuals with current or past cardiovascular, liver, renal, or pancreatic disease, malignancy, pregnancy or lactation, or regular use of prescribed medications for conditions other than hypertension (e.g., lipid-lowering or glucose-lowering agents for cardiometabolic diseases) were excluded based on the same questionnaire. This restriction was intended to minimize pharmacologic modification of key metabolic and inflammatory biomarkers, which could otherwise distort biomarker distributions and impair interpretability in a cross-sectional model-development setting. Importantly, exclusion was based on disease history and medication use rather than laboratory thresholds; thus, abnormal biomarker values could still be present and may reflect undiagnosed or untreated metabolic abnormalities, subclinical disease states, or biological variability inherent to single cross-sectional measurements.

### 2.2. Laboratory Assessments

Laboratory assessments were performed as previously described [14]. Briefly, body mass index (BMI) and waist-to-hip ratio (WHR) were calculated from standard anthropometry. BP was measured three times after ≥20 min of seated rest, and the mean was used. Fasting blood and urine samples (12 h) were analyzed for lipid and glycemic profiles, inflammatory and oxidative stress biomarkers, and arterial stiffness, including triglycerides (TGs), high-density lipoprotein (HDL) cholesterol, low-density lipoprotein (LDL) cholesterol, hemoglobin A1c (HbA1c), high-sensitivity C-reactive protein (hs-CRP), oxidized low-density lipoprotein (ox-LDL), interleukin (IL)-6, IL-1β, tumor necrosis factor-alpha (TNF-α), urinary 8-epi-prostaglandin F_2α_ (8-epi-PGF_2α_), plasma malondialdehyde (MDA), and brachial–ankle pulse wave velocity (ba-PWV). Insulin resistance (IR) was estimated using the homeostasis model assessment of insulin resistance (HOMA-IR): HOMA-IR = [fasting insulin (μIU/mL) × fasting glucose (mmol/L)]/22.5.

### 2.3. Genotyping and QC

Genotyping of participants was performed using the Affymetrix Axiom™ KORV1.1-96 Array at DNA LINK, Inc. (Seoul, Republic of Korea) according to the manufacturer’s guidelines. Approximately 200 ng of genomic DNA was amplified and randomly fragmented into 25 to 125 base pair (bp) fragments. The initial amplification was conducted in a 40 μL reaction mixture, generating fragments ranging from 200 to 1100 bp after optimization. During the fragmentation, the amplified products were further cleaved into 20 to 50 bp segments and end-labeled with biotinylated nucleotides. Following hybridization, stringent washing procedures were employed to remove the nonspecific binding. Polymorphic nucleotides were detected through a multi-color ligation reaction performed on the array surface. The arrays were then imaged using the GeneTitan MC Instrument (Affymetrix, Santa Clara, CA, USA), and the images were analyzed with the Genotyping Console™ Software (version 4.2; Affymetrix, Santa Clara, CA, USA).

Genotype data were generated using the Korean Chip (K-CHIP), developed by the Center for Disease Genome Science (Cheongju, Republic of Korea) (4845-301, 3000-3031). In the original genotyping dataset, 2167 participants and 833,086 SNPs were available. Quality control (QC) was applied at both the sample and SNP levels. Samples were excluded from the analysis if they showed inconsistent sex information or if their genotype missing rate exceeded 10%. At the SNP level, variants were excluded if the minor allele frequency was <0.01, the missing rate exceeded 5%, or there was significant deviation from Hardy–Weinberg equilibrium (*p* < 0.001). In addition, SNPs in high linkage disequilibrium were pruned using PLINK (pairwise LD *r*^2^ ≥ 0.5, --indep-pairwise 250 50 0.5). After applying these sample- and SNP-level filters, 2159 participants and 606,216 SNPs remained. Following LD pruning, a set of 394,222 SNPs in 2159 participants was retained.

To obtain the primary analytic dataset used in the present study, extended QC procedures were further applied to address cryptic relatedness and population stratification. First, individuals with pairwise relatedness (PI_HAT > 0.125) were excluded using PLINK (--rel-cutoff 0.125). Second, principal component analysis (PCA) was performed on genome-wide SNP data, and genetic outliers with absolute *Z*-scores > 6 in any of the first four principal components (PC1–PC4) were removed. After these extended QC steps, 2075 unrelated participants and 394,222 SNPs remained. This quality-controlled dataset constituted the base analytic cohort for all subsequent GWAS, phenotype-based association analyses, GRS construction, and discrimination analyses, unless explicitly stated otherwise for model-specific complete-case evaluations.

### 2.4. SNP Selection and GRS Development

A two-stage analytic strategy was employed for SNP selection and GRS development. In the first stage, GWASs were conducted using systolic and diastolic BP as continuous traits to efficiently screen BP-associated genetic variants [15]. In the second stage, BP-screened variants were evaluated for association with guideline-defined hypertension to support downstream GRS construction and to ensure clinical relevance [16].

Systolic and diastolic BP (mmHg) were analyzed as continuous traits using the mean BP values obtained as described in Section 2.2. Because hypertension, defined as a binary phenotype, is susceptible to treatment- and threshold-related heterogeneity, continuous BP traits were used at the GWAS screening stage to preserve quantitative information and maximize statistical power [17,18]. Individuals receiving antihypertensive medication may present with normotensive readings despite underlying hypertension, whereas untreated individuals near diagnostic thresholds may be classified differently due to minor measurement variation [17]. Continuous BP traits are, therefore, less sensitive to such misclassification.

Under an additive genetic model, linear regression analyses were conducted separately for systolic and diastolic BP, with each BP trait as the dependent variable and SNP genotype (coded as 0, 1, or 2 copies of the effect allele) as the independent variable, adjusting for age and sex. The GWASs were implemented using PLINK version 1.9.

For the SNP selection in this moderate-sized cohort, a study-specific screening threshold of *p* < 1 × 10^−5^ was applied rather than a formal genome-wide significance level. This threshold balances false-positive control and variant prioritization in moderate-sized cohorts, where genome-wide significance is typically underpowered. This threshold was used to identify a manageable set of candidate BP-associated variants for downstream validation, rather than for definitive genetic inference. SNPs meeting this criterion in either the systolic BP or diastolic BP GWAS were considered BP-associated variants. In total, nine SNPs met this threshold and were selected for further investigation (Table S1, Panel A). To contextualize these GWAS-screened loci, prior evidence from the NHGRI–EBI GWAS Catalog and targeted PubMed searches is summarized in Table S1 (Panel B). We note that GWAS Catalog entries may originate from a related Korean health-screening dataset; thus, they are presented to support internal consistency rather than independent external replication. Genotype distributions, minor allele frequencies, and Hardy–Weinberg equilibrium *p* values for these SNPs are provided in Table S2.

Because antihypertensive therapy may attenuate BP-based genetic associations, BP-screened variants were subsequently evaluated using logistic regression models for guideline-defined hypertension. This validation step ensured that only variants demonstrating consistent associations with the clinically defined phenotype were carried forward for GRS construction.

Single-SNP logistic regression analyses were performed to assess the association between each candidate SNP and hypertension status. SNPs were retained for GRS construction if they met all of the following prespecified criteria: (i) statistical significance (*p* < 0.05) in both the crude and age- and BMI-adjusted models, (ii) consistent directionality under genotype-model checks, and (iii) alignment to a risk-increasing direction after defining the risk allele accordingly. Additive coding (0/1/2 risk-allele dosage) was used by default. For low-frequency variants with sparse risk-allele homozygotes, a carrier (dominant) coding scheme (0/1; non-carrier vs. carrier of ≥1 risk allele) was applied to improve estimate stability and interpretability; specifically, rs117559502, rs116861740, rs142983199, and rs62099117 were modeled using carrier coding.

The weighted GRS was calculated as the sum of SNP-specific contributions. For each retained SNP, the genotype was coded as risk-allele dosage (0/1/2) under additive coding, or as carrier status (0/1) for the four variants listed above, and multiplied by the corresponding SNP weight (β); these products were then summed across variants. SNP weights (β coefficients) were taken from the unadjusted single-SNP logistic regression model fitted under the same coding scheme (additive or carrier) used for that SNP in the GRS, thereby avoiding over-adjustment and preserving each variant’s direct contribution to hypertension risk.

### 2.5. Statistical Analysis

After extended genetic QC, including the exclusion of related individuals (PI_HAT > 0.125) and genetic outliers identified by PCA (PC1–PC4), the base analytic cohort comprised 2075 participants. This quality-controlled dataset served as the reference population for all descriptive analyses and regression-based phenotype association analyses, unless otherwise specified for model-specific complete-case evaluations.

All statistical analyses were performed using IBM SPSS Statistics version 26.0 (IBM Corp., Chicago, IL, USA) and RStudio (version 2025.05.0+496; Posit Software, PBC, Boston, MA, USA). Skewed continuous variables were natural log-transformed prior to regression analyses to improve distributional symmetry and reduce the influence of outliers. Ba-PWV was measured in cm/s. For descriptive analyses, ba-PWV was log-transformed to address right-skewness, with summary statistics back-transformed and presented on the original scale. For regression and receiver operating characteristic (ROC) analyses, ba-PWV was modeled on its original scale and linearly scaled by 100 cm/s (1 unit = 100 cm/s) to facilitate interpretation of effect estimates. The same quality-controlled and linearly 100 cm/s–scaled ba-PWV variable was used consistently across all regression and ROC analyses. No trimming or winsorization was applied to extreme but biologically plausible values.

Distributional characteristics of all study variables and variable-specific valid sample sizes are summarized in Table S3. A concise summary of missing *n* (%) for key predictors and model-level complete-case sample sizes (pattern across predictors) is additionally provided in Table S4. Analyses were restricted to participants with available data for the variables of interest, and missing values were not imputed. Complete-case analysis was chosen to avoid introducing additional model uncertainty from imputation under potentially unverifiable missing-at-random assumptions. Although complete-case analysis may reduce sample size, it avoids reliance on strong modeling assumptions regarding the joint distribution of partially observed predictors.

Between-group comparisons were conducted using independent *t*-tests for continuous variables and chi-square tests for categorical variables. Logistic regression models were used to estimate odds ratios (ORs) and 95% confidence intervals (CIs), with adjustment for relevant covariates, including age and BMI, as specified for each analysis.

Because ba-PWV and urinary 8-epi-PGF_2α_ were measured only in subsets of participants, analyses involving these biomarkers were conducted using complete-case datasets. Consequently, analytic sample sizes varied according to the predictors included. Specifically, stepwise logistic regression for non-genetic predictor screening was performed among participants with complete data on hypertension status, BMI, ba-PWV, and 8-epi-PGF_2α_ (*n* = 786). In this subset-defined complete-case dataset, sex-stratified stepwise screening would further reduce sample size within each subgroup and may yield unstable, data-driven predictor sets. Therefore, stepwise screening was performed in the total complete-case sample only to identify a stable and interpretable set of candidate non-genetic predictors and to maintain comparability across sexes. Sex-specific models were subsequently fitted and evaluated in males and females separately using these candidates.

Stepwise logistic regression (forward likelihood-ratio method) was used solely as a screening procedure to identify a parsimonious set of candidate predictors. Following this screening step, final discrimination models were constructed using multivariable logistic regression with all selected predictors entered simultaneously (ENTER method). Model-predicted probabilities derived from these final ENTER models were used for ROC curve analysis to evaluate discriminative performance and to estimate the AUC; stepwise screening results were not used for inferential reporting.

ROC analyses were performed using model-specific complete-case datasets, resulting in analytic sample sizes of 775 participants in the total sample, 382 males, and 397 females. Optimal cutoffs were determined using the Youden index within each model-specific ROC analysis and are presented descriptively. Because optimal thresholds are sample-dependent and may vary across populations, they were not separately optimism-corrected and require external validation before clinical application. Internal validation was conducted using 1000 bootstrap resamples with replacement. Bootstrap validation was applied only to the final fixed discrimination model for each group, and in each resample, the final model was refitted to estimate optimism in AUC (ΔAUC), yielding optimism-corrected AUCs (apparent AUC − ΔAUC). In addition to discrimination, calibration was assessed using calibration-in-the-large (intercept) and calibration slope, and visualized using calibration plots comparing predicted and observed risks. Overall performance was additionally summarized using the Brier score. Calibration intercepts, slopes, and Brier scores were also optimism-corrected using the same bootstrap resampling procedure, with 95% confidence intervals estimated from the bootstrap distribution. The stepwise screening procedure was not repeated within each bootstrap resample. Accordingly, optimism estimates reflect the performance of the final prespecified models rather than full selection-process uncertainty. All statistical tests were two-tailed, with *p* < 0.05 considered statistically significant for hypothesis-driven analyses.

## 3. Results

### 3.1. Comparison of Baseline Characteristics Between Males and Females

After exclusion of related individuals and genetic outliers identified through identity-by-descent and principal component analyses (PCAs), the analytic cohort comprised 2075 unrelated participants (849 males and 1226 females), of whom 529 (25.5%) had hypertension (32.6% in males and 20.6% in females, *p* < 0.001). Males were slightly younger and had higher adiposity indices (BMI and waist circumference) and higher BP (all *p*s < 0.001). Sex differences were also observed in lipid profiles and inflammatory, oxidative stress, and arterial stiffness markers (Table 1). This pronounced sex difference in hypertension prevalence and cardiometabolic profiles provides essential epidemiological context for subsequent sex-stratified analyses and the interpretation of hypertension discrimination models.

### 3.2. Association of Anthropometric and Biochemical Variables with Prevalent Hypertension

Table 2 summarizes the association between anthropometric and biochemical variables and prevalent hypertension in the total sample and in sex-stratified subgroups. As expected, given the case definition, systolic and diastolic BP showed strong associations with prevalent hypertension. These BP associations are presented for descriptive completeness but were not considered for subsequent discrimination model building.

Across all groups (total sample, males, and females), higher urinary 8-epi-PGF_2α_ and greater ba-PWV were consistently and significantly associated with increased hypertension risk (all *p*s < 0.05).

In the total sample, higher body weight, fasting blood glucose, TGs, TNF-α, and IL-6 were positively associated with hypertension risk, whereas total cholesterol, HDL cholesterol, and LDL cholesterol showed inverse associations (all *p*s < 0.05). In males, body weight, waist circumference, fasting blood glucose, and TGs were positively associated with hypertension risk, while LDL cholesterol and IL-1β levels were inversely associated (all *p*s < 0.05). In females, inverse associations were observed between waist circumference, LDL cholesterol, and MDA and hypertension risk, whereas TNF-α was positively associated with hypertension (all *p*s < 0.05). An inverse association was also observed for waist-to-hip ratio (WHR; modeled per 0.1 increase); however, this finding should be interpreted cautiously given the limited variability of WHR and its substantial collinearity with other adiposity measures.

### 3.3. GRS Construction and Association with Hypertension

**Figure 1:**
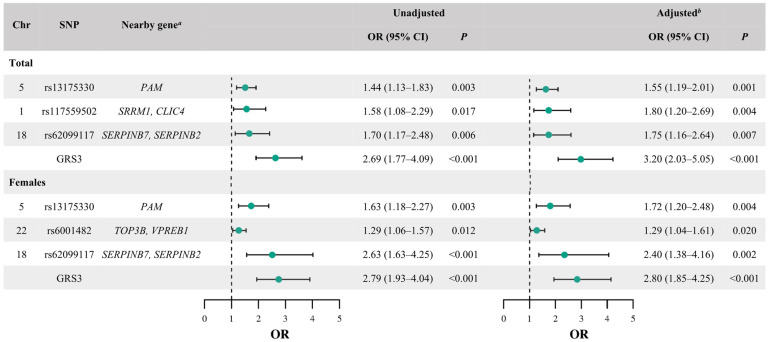
Association of genetic variants and genetic risk scores (GRSs) with hypertension risk. Odds ratios (ORs) and 95% confidence intervals (CIs) were derived from logistic regression analyses. Green dots indicate the OR point estimates, and the horizontal lines indicate the 95% CIs. *^a^* Unadjusted estimates are shown as reported in the original analysis. *^b^* Estimates adjusted for age and BMI are shown. The vertical dashed line indicates OR = 1 (no association). The GRS was constructed using prespecified selection criteria, including statistical significance (*p* < 0.05) in both the crude and age- and BMI-adjusted single-SNP logistic regression models, alignment of effect direction across genotype-model checks. SNP weights (β coefficients) were obtained from the corresponding unadjusted single-SNP logistic regression models. Genotype coding followed Table S5 (carrier coding for rs117559502 and rs62099117; additive coding for rs13175330 and rs6001482). Chr, chromosome; SNP, single-nucleotide polymorphism.

Based on the nine BP-related SNPs identified through the GWAS screening step, SNPs showing statistically significant associations and consistent effect directions were further evaluated for GRS construction (Figure 1). In the total sample, seven SNPs (rs13175330, rs1915872, rs6001482, rs12539814, rs4857055, rs117559502, and rs62099117) were significantly associated with hypertension in both unadjusted and age- and BMI-adjusted single-SNP logistic regression models (Table S5).

Applying the prespecified GRS selection criteria—statistical significance (*p* < 0.05) in both the crude and adjusted models, alignment of effect alleles to a risk-increasing direction, and consistency across genotype-model checks—three SNPs (rs13175330, rs117559502, and rs62099117) were retained and incorporated into GRS3 (Total). GRS3 (Total) showed a stronger association with prevalent hypertension than any individual SNP, with an unadjusted odds ratio (OR) of 2.69 (95% confidence interval [CI] = 1.77–4.09, *p* < 0.001) and an adjusted OR of 3.20 (95% CI = 2.03–5.05, *p* < 0.001) (Figure 1).

In the male subgroup, no SNP met the prespecified significance criteria in the unadjusted analyses, and only rs13175330 showed a nominal association after adjustment (*p* = 0.047); therefore, a male-specific GRS was not constructed. In the female group, four SNPs (rs13175330, rs6001482, rs4857055, and rs62099117) remained significant in both crude and adjusted models (Table S5). Using the same selection criteria, three SNPs (rs13175330, rs6001482, and rs62099117) were retained to construct GRS3 (Females). GRS3 (Females) demonstrated a stronger association with prevalent hypertension than individual SNPs, with an unadjusted OR of 2.79 (95% CI: 1.93–4.04, *p* < 0.001) and an adjusted OR of 2.80 (95% CI: 1.85–4.25, *p* < 0.001) (Figure 1).

### 3.4. Optimal Discriminators and Cutoff Values for Hypertension

Stepwise logistic regression in the total sample (forward likelihood-ratio method) was used solely as a screening procedure to identify key non-genetic discriminators of prevalent hypertension. This screening step was conducted among complete cases with non-missing hypertension status, BMI, ba-PWV, and 8-epi-PGF_2α_ (*n* = 786; Table S6) and identified BMI, ba-PWV, and 8-epi-PGF_2α_ as candidate predictors. The discriminative performance of these variables, individually and in combination with GRSs where applicable, was subsequently evaluated using multivariable logistic regression–based receiver operating characteristic (ROC) curve analysis in the total and in sex-specific subgroups (Table 3). ROC analyses were performed using model-specific complete-case datasets; therefore, analytic sample size varied by group and model.

**Figure 2:**
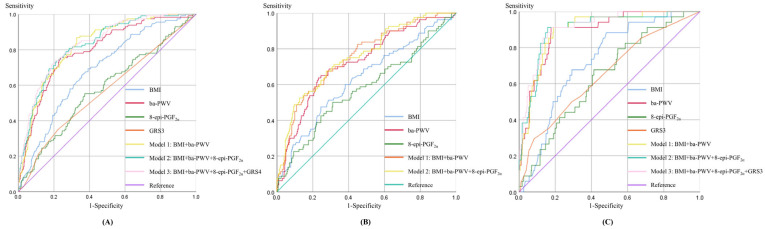
ROC curves for prevalent hypertension discrimination. (**A**) ROC curve for the total study population. (**B**) ROC curve for the male group. (**C**) ROC curve for the female group. Single-marker curves are labeled by predictor name; Model 1–3 correspond to the multivariable models defined in Table 3 (Model 1: BMI + ba-PWV; Model 2: BMI + ba-PWV + 8-epi-PGF_2α_; Model 3: BMI + ba-PWV + 8-epi-PGF_2α_ + GRS, where available). The diagonal reference line indicates no discrimination (AUC = 0.5). 8-epi-PGF_2α_, 8-epi-prostaglandin F_2α_; ba-PWV, Brachial–ankle pulse wave velocity; BMI, Body mass index; GRS, Genetic risk score; ROC, Receiver operating characteristic.

Among all groups, the female model combining BMI, ba-PWV, 8-epi-PGF_2α_, and GRS3 (Females) exhibited the highest discriminative performance, with an area under the curve (AUC) of 0.913 (95% CI: 0.869–0.956), a sensitivity of 91.2%, and a specificity of 82.1% in the complete-case female sample (*n* = 397) (Figure 2C). In the total sample (complete-case *n* = 775), the combined model including BMI, ba-PWV, 8-epi-PGF_2α_, and GRS3 (Total) also demonstrated strong discrimination (AUC = 0.833; 95% CI: 0.795–0.872) (Figure 2A). In males (complete-case *n* = 382), models incorporating only non-genetic predictors showed good discrimination, with AUCs of 0.754 for Model 1 (BMI + ba-PWV) and 0.758 for Model 2 (BMI + ba-PWV + 8-epi-PGF_2α_) (Figure 2B). No GRS met the prespecified stability and selection criteria in males after extended quality control (QC); therefore, no GRS was retained, and the final male discrimination models included only non-genetic predictors. This suggests potential sex-specific genetic architecture or limited statistical power for stable SNP selection in males.

Bootstrap internal validation using 1000 resamples yielded optimism-corrected AUCs of 0.827 (95% CI: 0.792–0.867) for the total model, 0.748 (95% CI: 0.688–0.805) for the male model, and 0.904 (95% CI: 0.865–0.948) for the female model (Table S7). These values were close to the corresponding apparent AUCs, indicating minimal apparent overfitting under internal validation. Optimism-corrected calibration intercepts were close to 0, and calibration slopes were near 1 in all groups, indicating overall acceptable calibration with no evidence of substantial miscalibration; calibration plots are provided in Figure S1, and Brier scores are reported in Table S7 as a summary of overall performance.

Optimal cutoff values were determined by maximizing the Youden index (Table 3). In the total sample, the optimal cutoffs were BMI = 23.9 kg/m^2^, ba-PWV = 1377.2 cm/s, 8-epi-PGF_2α_ = 1458.3 pg/mg creatinine (Cr), and GRS3 (Total) = 0.182. In males, the corresponding cutoffs were BMI = 25.6 kg/m^2^, ba-PWV = 1418.3 cm/s, and 8-epi-PGF_2α_ = 1452.9 pg/mg Cr. In females, the optimal cutoffs were BMI = 22.4 kg/m^2^, ba-PWV = 1385.3 cm/s, 8-epi-PGF_2α_ = 1484.9 pg/mg Cr, and GRS3 (Females) = 0.855. These thresholds are sample-derived and should be interpreted as descriptive estimates pending validation in independent populations.

## 4. Discussion

### 4.1. Sexual Dimorphism in Metabolic Factors

This study highlights a marked sexual dimorphism in cardiovascular metabolic profiles among Korean adults with and without prevalent hypertension. Male participants exhibited a more unfavorable baseline risk profile, characterized by higher BP, greater adiposity, increased arterial stiffness, and higher pro-inflammatory activity—factors that may contribute to an earlier or more pronounced manifestation of hypertension. In contrast, females exhibit a more complex and heterogeneous cardiovascular profile.

Elevated insulin levels observed in females may reflect sex-specific differences in glucose metabolism, potentially related to enhanced β-cell function and the multifaceted effects of estrogen. Estrogen has been shown to enhance insulin synthesis and secretion, augment incretin activity, suppress hepatic gluconeogenesis, and reduce systemic inflammation [19]. Although estrogen is also widely recognized for its antioxidant properties [20], females in the study showed higher levels of oxidized low-density lipoprotein (ox-LDL) and 8-epi-PGF_2α_. This finding is consistent with some prior reports suggesting greater oxidative burden in females [21], although results across studies remain inconsistent.

Such variability in reported sex differences in oxidative stress markers may arise from differences in marker selection, assay methodologies, and study populations [22]. Additionally, females demonstrated significantly higher levels of free fatty acids, total cholesterol, and LDL cholesterol levels, contrasting with the more classically described atherogenic lipid profiles in males [23]. These sex-specific lipid patterns may reflect the combined influence of hormonal fluctuations across reproductive stages, genetic factors—including those related to lipid metabolism on the X chromosome—and lifestyle-related determinants [24].

### 4.2. Sex-Specific Associations of Metabolic Factors with Prevalent Hypertension Risk

Building on the observed sex differences in metabolic profiles, subsequent analyses evaluated the sex-specific associations between these factors and prevalent hypertension risk. In the total population, established cardiometabolic factors–including measures of adiposity, glycemic status, lipid metabolism, oxidative stress, inflammation, and arterial stiffness–were significantly associated with prevalent hypertension in cross-sectional analyses, consistent with prior epidemiologic evidence [25,26,27].

Unexpectedly, total cholesterol and LDL cholesterol exhibited significant inverse associations with the odds of prevalent hypertension, contrary to conventional expectations [28]. In this cross-sectional setting, these counterintuitive associations likely reflect methodological and behavioral factors rather than a protective effect. Because eligibility was defined by medical history and medication use—not biomarker thresholds—untreated or subclinical dyslipidemia/dysglycemia could still be present within the analytic cohort. Moreover, the lipid–BP relationship may be non-linear across concentration ranges and subgroups [29], and residual confounding or reverse causality cannot be excluded (e.g., dietary or weight-control changes after hypertension awareness) [30,31]. Importantly, exclusion of individuals receiving lipid- and glucose-lowering therapy may introduce selection structures that preferentially remove participants with both high LDL cholesterol and treated hypertension, thereby altering lipid distributions and observed associations. Accordingly, these lipid findings should be interpreted cautiously and require confirmation in external and prospective cohorts.

In sex-stratified analyses, prevalent hypertension in males was positively associated with markers of obesity and metabolic burden, including body weight, waist circumference, TGs, and fasting glucose. In contrast, females demonstrated an inverse association between hypertension and waist circumference, consistent with sex-specific patterns of fat distribution. Females tend to accumulate a greater proportion of subcutaneous fat in the gluteofemoral region, which is considered metabolically less detrimental than the predominantly visceral fat distribution in males [32]. Supporting this interpretation, large-scale studies indicate that waist-related measures better predict hypertension in males, whereas BMI is more predictive in females [33]. An inverse association was also observed for WHR; however, this finding should be interpreted cautiously given the narrow physiological range of WHR and its substantial collinearity with other adiposity measures.

Inflammatory and oxidative stress markers also exhibited sex-specific associations with prevalent hypertension risk. In males, IL-1β showed a significant inverse association with hypertension, consistent with findings from a cross-sectional study in Swiss adults [34]. In females, TNF-α demonstrated a positive association, suggesting a more prominent role for TNF-α–mediated inflammation in hypertension among women, as supported by both animal and population studies [35,36]. Additionally, MDA exhibited an inverse association with hypertension risk exclusively in females, contradicting the established positive association between oxidative stress and hypertension [37]. However, sex-stratified evidence on this association remains limited, and this observation should be interpreted as exploratory.

### 4.3. Discriminative Roles of Key Metabolic Biomarkers in Hypertension

Given the pronounced sex-specific associations observed across metabolic factors, we next focused on identifying biomarkers with the greatest utility for discrimination and risk stratification of prevalent hypertension. Among the variables evaluated, three markers—BMI, ba-PWV, and 8-epi-PGF_2α_—showed the strongest discriminative performance across analyses. These biomarkers capture complementary dimensions of hypertension-related physiology, reflecting adiposity-related metabolic burden, arterial stiffness, and oxidative stress, respectively. Notably, all three exhibited sex-specific optimal cutoff values, supporting the use of sex-stratified approaches in hypertension discrimination.

BMI demonstrated discriminative value in both sexes, with a lower optimal cutoff in females (22.4 kg/m^2^) than in males (25.6 kg/m^2^), consistent with greater metabolic sensitivity at lower adiposity levels in women. For vascular function, sex-specific ba-PWV cutoffs were observed (1418.3 cm/s for males, 1385.3 cm/s for females). This pattern is consistent with prior evidence that arterial stiffness tends to manifest earlier or at higher absolute levels in males, whereas females may experience relative vascular protection before menopause, potentially related to estrogenic effects [36,38]. In contrast, the optimal cutoff for 8-epi-PGF_2α_ was higher in females (1484.9 pg/mg Cr) than in males (1452.9 pg/mg Cr), suggesting that a greater oxidative stress burden may be required before discrimination of prevalent hypertension in women. These findings should be interpreted within the context of cross-sectional discrimination rather than causal inference.

### 4.4. Genetic Variants Associated with Hypertension

In addition to the key metabolic predictors described above, genetic predisposition represents an important component of individual susceptibility to hypertension. In the present study, three SNPs were identified as significantly associated with prevalent hypertension in the total population. Prior evidence for the GWAS-screened loci (including those retained in the final GRS) is summarized in the NHGRI–EBI GWAS Catalog and targeted PubMed searches (Table S1B), supporting the internal consistency of our screening step (while not constituting independent external replication).

The SNP rs13175330 is located within an intronic region of the *PAM* gene, which encodes peptidylglycine α-amidating monooxygenase, a critical enzyme involved in the maturation of atrial natriuretic peptide (ANP) [39]. Altered PAM activity has been suggested to influence ANP-mediated vasodilation and natriuresis, thereby potentially contributing to BP regulation and hypertension risk [40]. Notably, the G allele of rs13175330 has previously been associated with increased hypertension risk in Korean populations [12]. The SNP rs117559502 is located near the *SRRM1* and *CLIC4* genes, which have been implicated in inflammatory signaling and oxidative stress regulation. *SRRM1* participates in Janus kinase/signal transducer and activator of transcription (JAK/STAT) signaling pathway, whereas *CLIC4* plays a role in maintaining endothelial redox homeostasis [41,42]. Lastly, rs62099117 lies adjacent to *SERPINB7* and *SERPINB2*, genes involved in renal fibrosis and inflammatory responses, respectively, suggesting a potential contribution of fibrotic and inflammatory mechanisms to hypertension susceptibility [43,44].

Importantly, the genetic screening strategy employed in this study aligns with prior hypertension genetics research, in which continuous BP traits are used at the initial GWAS stage to maximize statistical efficiency, followed by validation against clinically defined hypertension phenotypes to enhance translational relevance.

### 4.5. Sex-Specific Patterns of Genetic Risk for Hypertension

Sex-stratified analyses revealed differential patterns in genetic association with prevalent hypertension. In females, two SNPs identified in the total population (rs13175330 in *PAM* and rs62099117 near *SERPINB7/B2*) remained significantly associated with hypertension, suggesting relatively stronger or more detectable genetic effects in this subgroup. Additionally, a female-specific association was observed for rs6001482 near *TOP3B* and *VPREB1*, genes implicated in immune regulation, particularly B-cell-mediated immune processes [45,46]. Together, these findings are consistent with a potential contribution of immune-related pathways to BP regulation in females, although causal inferences cannot be drawn from the present cross-sectional analysis.

In contrast, no robust male-specific GRS could be constructed after application of prespecified selection and stability criteria. This likely reflects a combination of limited statistical power within the male subgroup and greater heterogeneity in genetic effects, such that the retained variants did not provide a sufficiently stable basis for score construction. Importantly, this observation does not indicate an absence of genetic contributions to hypertension in males; rather, under the current SNP set, sample size, and analytic framework, detectable and internally stable male genetic signals were insufficient to support a reliable weighted score.

Collectively, these results highlight sex-differential patterns in the genetic architecture of hypertension and underscore the importance of considering sex as a biological variable in genetic risk assessment and future hypertension research.

### 4.6. Sex-Specific Discrimination of Prevalent Hypertension Using Genetic and Clinical Markers

Following the identification of distinct sex-specific genetic and metabolic risk patterns, the combined discriminative value of these markers for hypertension was further evaluated. In the integrated models, genetic information provided modest but measurable improvements in discrimination in the total sample and in females, whereas the male model relied primarily on non-genetic predictors. Specifically, the model combining GRS with BMI, ba-PWV, and 8-epi-PGF_2α_ achieved good discrimination in females (AUC = 0.913) and in the total sample (AUC = 0.833). Bootstrap internal validation yielded optimism-corrected AUCs that were close to the apparent estimates (0.904 in females and 0.827 in the total sample), suggesting limited overfitting within the analyzed datasets.

In contrast, because no male-specific GRS met the prespecified stability and selection criteria, the final male discrimination model included non-genetic predictors (BMI, ba-PWV, and 8-epi-PGF_2α_), achieving an AUC of 0.758 (optimism-corrected AUC = 0.748). This pattern indicates that, within the constraints of the current SNP set and sample size, the incremental discriminative contribution of genetic information was more evident in females than in males. Such differences may reflect sex-specific genetic architecture, effect heterogeneity, as well as the limited internal stability of male genetic signals in this cohort.

Taken together, the present study establishes sex-specific discrimination models for prevalent hypertension using metabolic, vascular, oxidative and genetic markers, with optimized, sex-stratified cutoff values. Rather than serving as diagnostic tools to replace routine BP measurement, these models are intended to delineate sex-specific risk patterns and to provide an empirically derived framework for future prospective validation. The observed sex differences in optimal cutoffs and model composition underscore fundamental biological heterogeneity in hypertension pathophysiology and support the need for sex-aware approaches in risk stratification and preventive research.

### 4.7. Strengths and Limitations

To our knowledge, this study is among the first to systematically evaluate sex-stratified discrimination models for prevalent hypertension by integrating clinical biomarkers (BMI, ba-PWV, and 8-epi-PGF_2α_) with GRS, where stable SNP sets supported score construction (i.e., in the total sample and in females). By deriving sex-specific optimal cutoff values, the present work highlights differential contributions of metabolic, vascular, oxidative, genetic factors to hypertension across sexes. The use of routinely measured anthropometric indices in combination with mechanistically informative biomarkers provides translational insight and may inform risk stratification frameworks in similar health check-up settings, particularly in populations where sex-specific risk assessment tools remain limited. Moreover, because the data were collected prior to the COVID-19 pandemic, transportability to post-2020 populations may be limited, warranting external validation in post-COVID cohorts.

Nevertheless, several limitations deserve attention. First, the findings are derived from a single Korean cohort and require validation in independent populations and other ethnic groups to assess broader applicability. Although bootstrap internal validation was performed to reduce optimism in model performance estimates, external validation—preferably in prospective cohorts—is essential before broader interpretation.

Second, the cross-sectional design limits inference to prevalent hypertension, and temporality cannot be established. Some biomarkers may have been influenced by existing elevated BP and/or antihypertensive treatment, and reverse causality, therefore, cannot be excluded.

Third, key biomarkers such as ba-PWV and 8-epi-PGF_2α_ were available only in subsets of participants. Consequently, complete-case analyses were required, which may have reduced statistical power and limited generalizability, although this approach preserved internal consistency of model development under subset-based measurement constraints.

Fourth, residual confounding from unmeasured lifestyle or socioeconomic factors cannot be ruled out, and the limited availability of certain predictors (ba-PWV, 8-epi-PGF_2α_, and genetic testing) in routine clinical practice may constrain immediate clinical uptake. Additionally, our exclusion of individuals using lipid- or glucose-lowering medications yields a relatively untreated screening cohort, so performance and cutoffs may differ in medicated or clinically complex populations. Before applying these models beyond the current screening context, decision thresholds (including the Youden-derived cutoffs) should be re-evaluated and, if needed, recalibrated in the target population. Accordingly, proposed models should be viewed as proof-of-concept tools that provide a conceptual framework for understanding sex-specific contributions of genetic, vascular, and oxidative factors to hypertension risk, rather than as ready-to-implement diagnostic instruments.

Finally, although significant sex differences in genetic predictive performance were observed, the underlying molecular mechanisms remain to be elucidated. In particular, the absence of a stable male-specific GRS in this study likely reflects the limited incremental predictive value of the available SNP set beyond strong metabolic and vascular predictors under the current sample size and analytic framework, rather than an absence of genetic contributions to hypertension risk in males. Selection procedures and limited power may also have influenced the stability of male-specific SNP sets. Future studies incorporating larger samples and broader genetic instruments may help clarify the contexts in which genetic information provides additional value for sex-specific risk stratification.

## 5. Conclusions

In this study, we developed and internally evaluated sex-specific models for the discrimination of prevalent hypertension in a Korean population by integrating clinical biomarkers (BMI, ba-PWV, and 8-epi-PGF_2α_) with GRSs, where a stable score could be constructed. Across analyses, BMI, ba-PWV, and 8-epi-PGF_2α_ consistently demonstrated discriminative value, and the inclusion of GRS modestly improved model performance in the total sample and in females, with the most evident incremental contribution observed in females (AUC = 0.913).

The identification of sex-specific optimal cutoff values is consistent with biological heterogeneity in hypertension susceptibility and vascular–oxidative phenotypes between males and females, reinforcing the importance of sex-stratified approaches to risk stratification. Together, these findings suggest that integrating genetic information with metabolic and vascular markers may enhance the characterization of sex-specific risk patterns for hypertension.

At present, the proposed models should be regarded as proof-of-concept tools, as several components are not yet routinely available in primary care settings. Future studies are warranted to simplify these models using more widely accessible biomarkers and to validate their performance in independent, preferably prospective, cohorts and diverse ethnic populations to establish broader applicability and potential clinical utility.

## Figures and Tables

**Table 1:** Baseline characteristics of study participants stratified by sex: anthropometric, biochemical, and metabolic variables.

Variables	Valid *n* (Total/Males/Females)	Total (*n* = 2075)		Males (*n* = 849)		Females (*n* = 1226)		*p*
Age (years)	2075/849/1226	49.6	±0.25	48.5	±0.41	50.4	±0.31	<0.001
Weight (kg)	2073/849/1224	64.3	±0.24	71.7	±0.34	59.2	±0.24	<0.001
BMI (kg/m^2^)	2075/849/1226	24.1	±0.07	24.7	±0.10	23.8	±0.09	<0.001
Waist (cm)	2073/849/1224	84.6	±0.18	87.0	±0.25	83.0	±0.24	<0.001
WHR	2071/847/1224	0.89	±0.00	0.90	±0.00	0.88	±0.00	<0.001
Systolic BP (mmHg)	2075/849/1226	122.0	±0.35	126.0	±0.51	119.4	±0.45	<0.001
Diastolic BP (mmHg)	2075/849/1226	76.4	±0.25	78.9	±0.40	74.7	±0.31	<0.001
Glucose (mg/dL) *^∮^*	2074/849/1225	98.0	±0.50	102.3	±0.85	95.1	±0.59	<0.001
Insulin (μIU/mL) *^∮^*	2036/822/1214	9.25	±0.11	8.86	±0.17	9.51	±0.14	<0.001
HOMA-IR *^∮^*	2035/822/1213	2.25	±0.03	2.24	±0.06	2.25	±0.04	0.675
HbA1c (%) *^∮^*	598/256/342	6.14	±0.03	6.35	±0.06	5.98	±0.03	<0.001
Free fatty acids (μEq/L) *^∮^*	2009/819/1190	559.5	±5.69	512.2	±8.49	592.1	±7.48	<0.001
TGs (mg/dL) *^∮^*	2075/849/1226	126.9	±1.71	142.2	±3.03	116.3	±1.96	<0.001
Total cholesterol (mg/dL) *^∮^*	2075/849/1226	197.7	±0.79	192.8	±1.17	201.1	±1.06	<0.001
HDL cholesterol (mg/dL) *^∮^*	2075/849/1226	52.9	±0.30	48.7	±0.41	55.8	±0.39	<0.001
LDL cholesterol (mg/dL) *^∮^*	2049/832/1217	120.3	±0.73	116.7	±1.11	122.7	±0.95	<0.001
hs-CRP (mg/L) *^∮^*	1985/795/1190	1.29	±0.06	1.52	±0.12	1.13	±0.06	<0.001
MDA (nmol/mL) *^∮^*	1769/729/1040	9.14	±0.09	10.1	±0.13	8.48	±0.13	<0.001
ox-LDL (U/L) *^∮^*	1776/706/1070	46.3	±0.48	44.1	±0.72	47.8	±0.63	<0.001
8-epi-PGF_2α_ (pg/mg Cr) *^∮^*	1840/740/1100	1513.6	±20.0	1430.7	±30.8	1569.4	±26.2	0.001
TNF-α (pg/mL) *^∮^*	1577/630/947	10.9	±0.85	12.9	±2.00	9.53	±0.47	<0.001
IL-1β (pg/mL) *^∮^*	1605/640/965	0.90	±0.08	1.08	±0.20	0.79	±0.02	0.041
IL-6 (pg/mL) *^∮^*	1582/632/950	3.68	±0.10	4.07	±0.17	3.42	±0.12	<0.001
ba-PWV (cm/s) *^∮^*	802/389/413	1313.6	±7.08	1350.9	±9.62	1278.4	±10.0	<0.001
Hypertension, *n* (%)	2075/849/1226	529 (25.5)		277 (32.6)		252 (20.6)		<0.001

Analyses were conducted in 2075 unrelated participants after genetic quality control (QC) unless otherwise specified. Data are presented as the means ± standard errors (SEs) or as *n* (%), based on the valid number of observations for each variable. Valid sample sizes are reported as Total/Males/Females. *p*-values were calculated using an independent *t*-test for continuous variables and chi-square test for categorical variables. Variables marked with *^∮^* were natural log-transformed for between-group comparisons (including ba-PWV); descriptive statistics are shown on the original scale. ba-PWV and urinary 8-epi-PGF_2α_ were measured only in subsets of participants due to logistical constraints; therefore, valid sample sizes for these variables are smaller than the full analytic cohort. Variable transformations and rescaling applied in regression and discrimination analyses are described in the Statistical Analysis section. 8-epi-PGF_2α_, 8-epi-prostaglandin F_2α_; ba-PWV, brachial–ankle pulse wave velocity; BMI, body mass index; BP, blood pressure; Cr, creatinine; HbA1c, hemoglobin A1c; HDL, high-density lipoprotein; HOMA-IR, homeostasis model assessment—insulin resistance; hs-CRP, high-sensitivity C-reactive protein; IL, interleukin; LDL, low-density lipoprotein; MDA, malondialdehyde; ox-LDL, oxidized low-density lipoprotein; TGs, triglycerides; TNF-α, tumor necrosis factor-alpha; WHR, waist-to-hip ratio.

**Table 2:** Logistic regression analysis of hypertension risk based on anthropometric, biochemical, and metabolic variables in total, male, and female groups.

Variables	Total		Males		Females	
	OR (95% CI)	*p*	OR (95% CI)	*p*	OR (95% CI)	*p*
Age (years)	1.06 (1.05–1.07)	<0.001	1.04 (1.03–1.06)	<0.001	1.08 (1.07–1.10)	<0.001
Weight (kg)	1.05 (1.03–1.07)	<0.001	1.04 (1.00–1.07)	0.038	0.99 (0.95–1.03)	0.717
Waist (cm)	1.01 (0.99–1.03)	0.190	1.04 (1.01–1.08)	0.016	0.97 (0.94–0.99)	0.014
BMI (kg/m^2^)	1.22 (1.17–1.26)	<0.001	1.20 (1.13–1.27)	<0.001	1.22 (1.16–1.28)	<0.001
WHR (per 0.1 increase)	0.91 (0.74–1.12)	0.364	1.18 (0.82–1.69)	0.381	0.65 (0.50–0.85)	0.001
Systolic BP (mmHg)	1.14 (1.13–1.16)	<0.001	1.15 (1.13–1.17)	<0.001	1.13 (1.11–1.15)	<0.001
Diastolic BP (mmHg)	1.19 (1.17–1.22)	<0.001	1.19 (1.16–1.22)	<0.001	1.20 (1.17–1.23)	<0.001
Glucose (mg/dL) *^∮^*	2.78 (1.63–4.75)	<0.001	2.80 (1.31–6.01)	0.008	1.80 (0.80–4.07)	0.155
Insulin (μIU/mL) *^∮^*	0.93 (0.72–1.20)	0.577	1.00 (0.68–1.48)	0.988	1.06 (0.74–1.52)	0.741
HOMA-IR *^∮^*	1.15 (0.91–1.46)	0.242	1.17 (0.83–1.66)	0.370	1.14 (0.82–1.57)	0.432
HbA1c (%) *^∮^*	1.51 (0.29–7.77)	0.621	0.93 (0.13–6.60)	0.943	1.53 (0.07–32.1)	0.785
Free fatty acids (μEq/L) *^∮^*	1.24 (0.97–1.59)	0.090	1.12 (0.80–1.56)	0.516	1.36 (0.93–1.97)	0.109
TGs (mg/dL) *^∮^*	1.57 (1.28–1.92)	<0.001	1.52 (1.14–2.02)	0.004	1.31 (0.96–1.78)	0.085
Total cholesterol (mg/dL) *^∮^*	0.52 (0.29–0.92)	0.008	0.73 (0.32–1.65)	0.368	0.51 (0.22–1.17)	0.112
HDL cholesterol (mg/dL) *^∮^*	0.59 (0.39–0.90)	0.011	0.97 (0.51–1.84)	0.930	0.67 (0.36–1.25)	0.211
LDL cholesterol (mg/dL) *^∮^*	0.50 (0.34–0.72)	<0.001	0.56 (0.34–0.95)	0.030	0.51 (0.30–0.89)	0.017
hs-CRP (mg/L) *^∮^*	1.01 (0.92–1.11)	0.803	1.07 (0.93–1.23)	0.370	0.94 (0.82–1.07)	0.367
MDA (nmol/mL) *^∮^*	1.10 (0.79–1.54)	0.563	1.24 (0.76–2.05)	0.419	0.59 (0.36–0.98)	0.042
ox-LDL (U/L) *^∮^*	0.99 (0.75–1.32)	0.951	0.92 (0.61–1.37)	0.667	1.29 (0.85–1.95)	0.237
8-epi-PGF_2α_ (pg/mg Cr) *^∮^*	1.74 (1.38–2.20)	<0.001	1.61 (1.13–2.29)	0.005	2.08 (1.50–2.87)	<0.001
TNF-α (pg/mL) *^∮^*	1.22 (1.06–1.40)	0.007	1.09 (0.89–1.35)	0.407	1.24 (1.02–1.52)	0.031
IL-1β (pg/mL) *^∮^*	0.96 (0.81–1.15)	0.654	0.76 (0.58–0.99)	0.046	1.10 (0.86–1.41)	0.446
IL-6 (pg/mL) *^∮^*	1.19 (1.02–1.38)	0.029	1.07 (0.86–1.35)	0.537	1.19 (0.96–1.47)	0.105
ba-PWV (scaled; 1 unit = 100 cm/s)	1.53 (1.35–1.75)	<0.001	1.42 (1.21–1.67)	<0.001	1.65 (1.29–2.11)	<0.001

Analyses were conducted in 2075 unrelated participants after genetic QC unless otherwise specified. Data are presented as odds ratios (ORs) and 95% confidence intervals (CIs). For each variable, a separate age- and BMI-adjusted logistic regression model was fitted in the total sample and stratified by sex, using participants with non-missing data for that variable. The age model was adjusted for BMI only, and the BMI model was adjusted for age only. WHR was modeled per 0.1 increase to improve interpretability given its narrow physiological range and substantial collinearity with other adiposity measures, and ba-PWV was scaled by 100 cm/s (1 unit = 100 cm/s). Variables marked with *^∮^* were natural log-transformed (ln) before modeling. Systolic and diastolic BP are presented for descriptive completeness, given the case definition, and were not considered for subsequent discrimination model building. *p*-values were obtained from the corresponding adjusted logistic regression models. Differences in valid sample sizes across variables reflect laboratory measurement availability rather than participant exclusion. 8-epi-PGF_2α_, 8-epi-prostaglandin F_2α_; ba-PWV, Brachial–ankle pulse wave velocity; BP, blood pressure; Cr, creatinine; HbA1c, hemoglobin A1c; HDL, high-density lipoprotein; HOMA-IR, homeostasis model assessment—insulin resistance; hs-CRP, high-sensitivity C-reactive protein; IL, interleukin; LDL, low-density lipoprotein; MDA, malondialdehyde; ox-LDL, oxidized low-density lipoprotein; TGs, triglycerides; TNF-α, tumor necrosis factor-alpha; WHR, waist-to-hip ratio.

**Table 3:** Discriminative performance of the hypertension classification models.

	Cutoff Value	Sensitivity (%)	Specificity (%)	AUC	95% CI	*p*
**Total (*****n*** **= 775)**						
BMI	23.9	69.3	60.8	0.688	0.638–0.737	<0.001
ba-PWV	1377.2	74.6	77.0	0.798	0.754–0.841	<0.001
8-epi-PGF_2α_	1458.3	55.3	62.6	0.578	0.520–0.637	<0.001
GRS3	0.182	35.1	77.6	0.565	0.506–0.625	0.007
Model 1	0.115	86.8	67.2	0.826	0.789–0.863	<0.001
Model 2	0.159	73.7	78.7	0.827	0.789–0.866	<0.001
Model 3	0.169	71.9	81.1	0.833	0.795–0.872	<0.001
**Males (*****n*** **= 382)**						
BMI	25.6	47.5	75.5	0.631	0.560–0.702	<0.001
ba-PWV	1418.3	63.8	76.8	0.726	0.663–0.789	<0.001
8-epi-PGF_2α_	1452.9	50.0	68.9	0.577	0.503–0.652	0.033
Model 1	0.219	67.5	71.9	0.754	0.695–0.814	<0.001
Model 2	0.221	67.5	72.5	0.758	0.698–0.817	<0.001
**Females (*****n*** **= 397)**						
BMI	22.4	88.2	51.8	0.724	0.650–0.799	<0.001
ba-PWV	1385.3	91.2	82.4	0.891	0.841–0.941	<0.001
8-epi-PGF_2α_	1484.9	67.6	58.7	0.639	0.549–0.729	0.007
GRS3	0.855	29.4	91.7	0.651	0.552–0.750	0.004
Model 1	0.078	91.2	81.0	0.900	0.853–0.946	<0.001
Model 2	0.092	91.2	84.3	0.901	0.848–0.955	<0.001
Model 3	0.071	91.2	82.1	0.913	0.869–0.956	<0.001

Discrimination analyses were conducted in quality-controlled, unrelated participants using model-specific complete-case datasets defined by the availability of predictors included in each model. *p*-values were derived from receiver operating characteristic (ROC) curve analysis testing H0: AUC = 0.5. AUCs were calculated using model-predicted probabilities obtained from multivariable logistic regression models and are invariant to linear rescaling or monotonic transformation of predictors. Cutoff values were determined by maximizing the Youden index. Model 1: BMI + ba-PWV; Model 2: BMI + ba-PWV + 8-epi-PGF_2α_; Model 3: BMI + ba-PWV + 8-epi-PGF_2α_ + GRS (when available; no GRS met the prespecified stability and selection criteria in males). For single-marker analyses, ba-PWV was scaled by 100 cm/s (1 unit = 100 cm/s) and urinary 8-epi-PGF_2α_ was ln-transformed; cutoff values are reported on the original scales (ba-PWV in cm/s after multiplying by 100; 8-epi-PGF_2α_ in pg/mg creatinine after back-transformation). The ba-PWV variable refers to the same quality-controlled and 100 cm/s–scaled measure used consistently across Table 2 and Supplementary Tables S6 and S7. 8-epi-PGF_2α_, 8-epi-prostaglandin F_2α_; AUC, Area under the curve; ba-PWV, Brachial–ankle pulse wave velocity; BMI, Body mass index; CI, Confidence interval; GRS, Genetic risk score.

## Data Availability

The data presented in this study are available on request from the corresponding author. The data are not publicly available due to privacy or ethical restrictions.

## Supplementary Materials

The following supporting information can be downloaded at https://www.mdpi.com/article/10.3390/cimb48030271/s1.

## Abbreviations

The following abbreviations are used in this manuscript:

8-epi-PGF_2α_	8-epi-prostaglandin F_2α_
ANP	Atrial natriuretic peptide
AUC	Area under the curve
ba-PWV	Brachial–ankle pulse wave velocity
BMI	Body mass index
BP	Blood pressure
CI	Confidence interval
Cr	Creatinine
GRS	Genetic risk score
GWAS	Genome-wide association study
HbA1c	Hemoglobin A1c
HDL	High-density lipoprotein
HOMA	Homeostasis model assessment
Hs-CRP	High-sensitivity C-reactive protein
IL	Interleukin
IR	Insulin resistance
K-CHIP	Korean chip
LDL	Low-density lipoprotein
MDA	Malondialdehyde
OR	Odds ratio
Ox-LDL	Oxidized low-density lipoprotein
ROC	Receiver operating characteristic
SNP	Single-nucleotide polymorphism
TGs	Triglycerides
TNF-α	Tumor necrosis factor-alpha
WHR	Waist-to-hip ratio
