# Supplementary material for: Development of a Sex-Specific Prevalent Hypertension Discrimination Model in Korean Adults Using Genetic Risk Scores and Clinical Biomarkers: A Cross-Sectional Study
Source: Curr Issues Mol Biol. 2026 Mar 3;48(3):271. doi: 10.3390/cimb48030271 (PMC13025723; doi:10.3390/cimb48030271)
Supplement: Supplementary file 1 [file cimb-48-00271-s001.zip › cimb-4165863-supplementary.pdf]

Table S1. GWAS screening results for the nine BP-associated SNPs after extended QC (Panel A) and prior evidence from the NHGRI-EBI GWAS Catalog / literature (Panel B)

Panel A. GWAS screening results for BP-associated SNPs after extended QC

No.	Chr	SNP	Nearby gene[a]	Primary BP trait[b]	Systolic BP		Diastolic BP	
					β	*P*	β	*P*
1	5	rs13175330	*PAM*	Diastolic BP	3.499	3.47×10^{-5}	2.972	1.72×10^{-6}
2	1	rs1915872	*RGS7*	Diastolic BP	2.086	7.65×10^{-4}	2.219	1.09×10^{-6}
3	22	rs6001482	*TOP3B, VPREB1*	Diastolic BP	1.696	2.68×10^{-4}	1.735	3.84×10^{-7}
4	7	rs12539814	*NOBOX*	Diastolic BP	-1.756	2.44×10^{-4}	-1.587	6.49×10^{-6}
5	3	rs4857055	*EPHA6*	Systolic BP	2.473	1.19×10^{-7}	1.332	1.10×10^{-4}
6	1	rs117559502	*SRRM1, CLIC4*	Systolic BP	6.212	2.71×10^{-6}	3.012	2.02×10^{-3}
7	21	rs116861740	*LINC00478, C21orf37*	Systolic BP	5.642	9.55×10^{-6}	1.408	1.34×10^{-1}
8	12	rs142983199	*AMN1*	Systolic BP	7.525	6.71×10^{-6}	3.727	2.48×10^{-3}
9	18	rs62099117	*SERPINB7, SERPINB2*	Systolic BP	6.308	3.20×10^{-6}	2.748	5.98×10^{-3}

Panel B. Prior evidence for the nine GWAS-screened BP loci (NHGRI-EBI GWAS Catalog / literature)

No.	SNP	Prior evidence source	Reported phenotype/trait	GWAS Catalog accession	Peer-reviewed BP/HTN evidence
1	rs13175330	GWAS Catalog + Literature	Systolic / Diastolic BP	GCST005169; GCST005168	Yes (PMID: 29162152)
2	rs1915872	GWAS Catalog	Diastolic BP	GCST005169	No
3	rs6001482	GWAS Catalog	Diastolic BP	GCST005169	No
4	rs12539814	GWAS Catalog	Diastolic BP	GCST005169	No
5	rs4857055	GWAS Catalog + Literature	Systolic BP	GCST005168	Yes (PMID: 29208002)
6	rs117559502	GWAS Catalog	Systolic BP	GCST005168	No
7	rs116861740	GWAS Catalog	Systolic BP	GCST005168	No
8	rs142983199	GWAS Catalog	Systolic BP	GCST005168	No
9	rs62099117	GWAS Catalog	Systolic BP	GCST005168	No

Panel A. GWAS was performed in 2,075 participants after extended QC. [a]Information in the original report is shown. [b]"Primary BP trait" denotes whether the SNP was originally selected from the GWAS of systolic or diastolic BP using the threshold $P < 1\times10^{-5}$. BP, Blood pressure; Chr, Chromosome; GWAS, Genome-wide association study; No., Number; QC, quality control; SNP, Single-nucleotide polymorphism.

Panel B summarizes prior evidence for the nine SNPs based on the NHGRI–EBI GWAS Catalog and PubMed searches. Catalog entries support consistency with previously reported BP loci; however, the cataloged study may include overlapping participants from a related Korean health-screening dataset. Therefore, these entries should be interpreted as supporting *internal consistency* rather than independent external replication.

Table S2. Genotype distributions and quality metrics (MAF and HWE) for the nine SNPs

No.	Chr	SNP	Nearby gene[a]	Genotype[a]	Valid *n*	Genotype counts, *n* (0 / 1 / 2 risk alleles)[b]	MAF	HWE *P*
**1**	**5**	**rs13175330**	***PAM***	**A>G**	**2,069**	**1,739 / 318 / 12**	**0.082**	**0.663**
2	1	rs1915872	*RGS7*	G>A	2,022	1,399 / 561 / 62	0.169	0.527
**3**	**22**	**rs6001482**	***TOP3B, VPREB1***	**A>G**	2,074	**697 / 1,003 / 374**	**0.422**	**0.686**
4	7	rs12539814	*NOBOX*	A>G	2,075	822 / 973 / 280	0.369	0.814
5	3	rs4857055	*EPHA6*	C>T	2,065	562 / 1,046 / 457	0.475	0.508
**6**	**1**	**rs117559502**	***SRRM1, CLIC4***	**T>C**	**2,073**	**1,942 / 130 / 1**	**0.032**	**0.721**
7	21	rs116861740	*LINC00478, C21orf37*	G>C	2,070	1,927 / 142 / 1	0.035	0.515
8	12	rs142983199	*AMN1*	C>T	2,072	1,990 / 82 / 0	0.020	1.000
**9**	**18**	**rs62099117**	***SERPINB7, SERPINB2***	**A>T**	**2,055**	**1,927 / 128 / 0**	**0.031**	**0.263**

[a]Information in the original report is shown. [b]Genotype counts are presented as the numbers of individuals carrying 0, 1, and 2 copies of the coded allele (A1) in the PLINK output. SNPs in bold were included in the construction of the genetic risk scores (GRS) used in the prediction models.
Chr, Chromosome; HWE, Hardy–Weinberg equilibrium; MAF, Minor allele frequency; No., Number; SNP, Single-nucleotide polymorphism.

Table S3. Distributional characteristics of study variables

Variable	Group	Valid *n*	Min	Q1 (25th)	Median (50th)	Q3 (75th)	Max	IQR (Q3–Q1)
Age (years)	Total	2,075	20.00	41.00	50.00	58.00	86.00	17.00
	Males	849	20.00	40.00	50.00	58.00	77.00	18.00
	Females	1,226	21.00	42.00	51.00	58.00	86.00	16.00
Weight (kg)	Total	2,073	25.00	56.40	63.30	71.20	119.60	14.80
	Males	849	25.00	65.40	71.00	77.40	119.60	12.10
	Females	1,224	33.00	53.80	58.30	64.20	96.80	10.40
BMI (kg/m²)	Total	2,075	14.20	22.10	24.10	26.00	38.20	3.90
	Males	849	16.70	22.80	24.60	26.30	38.20	3.50
	Females	1,226	14.20	21.60	23.50	25.70	34.90	4.10
Waist (cm)	Total	2,073	57.50	79.20	84.50	90.00	119.00	10.90
	Males	849	66.00	82.00	86.50	91.50	119.00	9.50
	Females	1,224	57.50	77.00	83.00	88.00	110.00	11.00
WHR	Total	2,071	0.65	0.85	0.89	0.93	1.27	0.08
	Males	847	0.75	0.87	0.90	0.93	1.27	0.06
	Females	1,224	0.65	0.84	0.89	0.93	1.09	0.09
Systolic BP (mmHg)	Total	2,075	79.50	110.00	121.00	132.00	206.50	22.00
	Males	849	87.50	115.50	125.00	135.00	206.50	19.80
	Females	1,226	79.50	107.50	117.50	129.00	198.00	21.50
Diastolic BP (mmHg)	Total	2,075	32.50	69.00	76.00	84.00	120.00	15.00
	Males	849	32.50	71.00	78.00	86.00	120.00	15.00
	Females	1,226	36.00	67.00	74.00	82.00	110.00	15.00
Glucose (mg/dL)	Total	2,074	54.00	85.00	93.00	104.00	302.00	19.00
	Males	849	54.00	88.00	96.00	109.00	302.00	21.00
	Females	1,225	59.00	84.00	91.00	101.00	285.00	17.00
Insulin (µIU/mL)	Total	2,036	1.00	6.35	8.20	10.80	80.30	4.45
	Males	822	1.20	6.00	7.80	10.30	65.40	4.30
	Females	1,214	1.00	6.60	8.50	11.20	80.30	4.60
HOMA-IR	Total	2,035	0.17	1.47	1.93	2.60	28.60	1.13
	Males	822	0.30	1.44	1.88	2.56	28.60	1.13
	Females	1,213	0.17	1.48	1.95	2.62	22.60	1.14
HbA1c (%)	Total	598	4.80	5.70	6.00	6.40	12.30	0.70
	Males	256	4.90	5.80	6.20	6.60	12.30	0.80
	Females	342	4.80	5.60	5.80	6.20	8.80	0.60
Free fatty acids (µEq/L)	Total	2,009	71.00	386.00	522.00	691.50	2784.00	305.50
	Males	819	79.00	348.00	469.00	627.50	2784.00	279.50
	Females	1,190	71.00	413.00	546.50	727.00	2186.00	314.00
Triglycerides (mg/dL)	Total	2,075	15.00	74.00	107.00	159.00	915.00	85.00
	Males	849	15.00	84.00	119.00	173.00	915.00	89.00
	Females	1,226	22.00	68.00	101.00	145.00	588.00	77.00
Total cholesterol (mg/dL)	Total	2,075	48.00	173.00	196.00	219.00	408.00	46.00
	Males	849	48.00	170.00	191.00	214.00	408.00	44.00
	Females	1,226	110.00	176.00	200.00	224.00	342.00	48.00
HDL cholesterol (mg/dL)	Total	2,075	20.00	43.00	51.00	61.00	120.00	18.00
	Males	849	20.00	41.00	47.00	55.00	120.00	14.00
	Females	1,226	20.00	46.00	55.00	65.00	114.00	19.00

LDL cholesterol (mg/dL)	Total	2,049	21.60	98.20	118.20	141.00	337.80	42.80
	Males	832	21.60	95.50	115.30	136.90	337.80	41.50
	Females	1,217	38.40	100.40	121.00	142.80	249.20	42.50
hs-CRP (mg/L)	Total	1,985	0.01	0.29	0.57	1.20	49.20	0.91
	Males	795	0.01	0.36	0.67	1.31	49.20	0.95
	Females	1,190	0.01	0.25	0.52	1.12	29.80	0.87
MDA (nmol/mL)	Total	1,769	0.62	7.01	8.39	10.30	81.40	3.32
	Males	729	0.88	7.86	9.54	11.40	34.90	3.60
	Females	1,040	0.62	6.63	7.80	9.34	81.40	2.71
ox-LDL (U/L)	Total	1,776	8.49	31.80	42.90	56.30	198.00	24.60
	Males	706	12.40	30.20	40.30	53.80	144.70	23.60
	Females	1,070	8.49	33.20	44.10	57.90	198.00	24.80
8-epi-PGF$_{2\alpha}$ (pg/mg Cr)	Total	1,840	49.20	980.80	1323.20	1811.40	11986.40	830.90
	Males	740	285.70	918.70	1250.50	1692.40	11986.40	773.80
	Females	1,100	49.20	1016.70	1372.10	1898.20	9001.80	882.80
TNF-α (pg/mL)	Total	1,577	0.00	4.49	7.44	11.30	1193.30	6.80
	Males	630	0.00	5.14	8.35	12.00	1193.30	6.81
	Females	947	0.00	4.08	6.81	11.10	312.90	7.06
IL-1β (pg/mL)	Total	1,605	0.00	0.42	0.60	0.97	126.10	0.55
	Males	640	0.00	0.43	0.60	0.97	126.10	0.54
	Females	965	0.00	0.42	0.60	0.96	6.45	0.54
IL-6 (pg/mL)	Total	1,582	0.03	1.69	2.83	4.32	55.70	2.63
	Males	632	0.05	2.02	3.23	4.64	55.70	2.63
	Females	950	0.03	1.58	2.54	4.12	53.60	2.55
ba-PWV (cm/s)	Total	802	908.50	1180.00	1286.30	1421.00	2426.00	241.10
	Males	389	908.50	1224.50	1327.00	1463.50	2406.50	241.80
	Females	413	909.00	1145.00	1242.00	1376.00	2426.00	231.30

Data are presented as minimum (Min), 25th percentile (Q1), median (Q2), 75th percentile (Q3), maximum (Max), and interquartile range (IQR = Q3–Q1). Valid *n* indicates the number of participants with non-missing values for each variable; some biomarkers (e.g., HbA1c, inflammatory/oxidative stress markers, and ba-PWV) were available only in subsets due to laboratory or measurement availability.

8-epi-PGF$_{2\alpha}$, 8-epi-prostaglandin F$_{2\alpha}$; ba-PWV, Brachial-ankle pulse wave velocity; BP, Blood pressure; Cr, Creatinine; HbA1c, Hemoglobin A1c; HDL, High-density lipoprotein; HOMA-IR, Homeostasis model assessment-insulin resistance; hs-CRP, High-sensitivity C-reactive protein; IL, Interleukin; LDL, Low-density lipoprotein; MDA, Malondialdehyde; ox-LDL, Oxidized low-density lipoprotein; TGs, Triglycerides; TNF-α, Tumor necrosis factor-alpha; WHR, Waist-to-hip ratio.

Table S4. Extent and pattern of missing data for key predictors used in the discrimination models

Panel A. Predictor-level missingness (variable-specific)

Population	N (denominator)	Predictor	Valid *n*	Missing *n* (%)
Total	2,075	ba-PWV	802	1,273 (61.3)
		8-epi-PGF$_{2α}$	1,840	235 (11.3)
		GRS3 (Total)	2,049	26 (1.3)
Males	849	ba-PWV	389	460 (54.2)
		8-epi-PGF$_{2α}$	740	109 (12.8)
Females	1,226	ba-PWV	413	813 (66.3)
		8-epi-PGF$_{2α}$	1,100	126 (10.3)
		GRS3 (Females)	1,209	17 (1.4)

Panel B. Model-level complete-case sample sizes (pattern across predictors)

Analysis step / model	Required variables	Complete-case N	% of denominator
Stepwise screening (total sample)	HTN + BMI + ba-PWV + 8-epi-PGF$_{2α}$	786	37.9% of 2,075
ROC (Total Model 3)	HTN + BMI + ba-PWV + 8-epi-PGF$_{2α}$ + GRS3 (Total)	775	37.3% of 2,075
ROC (Male Model 2)	HTN + BMI + ba-PWV + 8-epi-PGF$_{2α}$	382	45.0% of 849
ROC (Female Model 3)	HTN + BMI + ba-PWV + 8-epi-PGF$_{2α}$ + GRS3 (Females)	397	32.4% of 1,226

Missingness primarily reflects subset-based availability of ba-PWV and urinary 8-epi-PGF$_{2α}$ measurements rather than participant exclusion. No imputation was performed. Denominators correspond to the QC-passed analytic cohort (total *n* = 2,075; males *n* = 849; females *n* = 1,226). Hypertension status and BMI had no missing values in the analytic cohort; therefore, Panel A focuses on predictors with non-zero missingness.

8-epi-PGF$_{2α}$, 8-epi-prostaglandin F$_{2α}$; ba-PWV, Brachial-ankle pulse wave velocity; BMI, body mass index; GRS, genetic risk score; HTN, hypertension; QC, quality control; ROC, receiver operating characteristic.

Table S5. Association of genetic variants and genetic risk scores (GRS) with hypertension risk

No.	Chr	SNP	Nearby gene[a]	Genotype[a]	Valid *n*	Unadjusted			Adjusted[b]		
						OR (95% CI)	*β*	*P*	OR (95% CI)	*β*	*P*
**Total (*n* = 2,075)**											
**1**	**5**	**rs13175330**	***PAM***	**A>G**	**2,069**	**1.44 (1.13–1.83)**	**0.363**	**0.003**	**1.55 (1.19–2.01)**	**0.438**	**0.001**
2	1	rs1915872	*RGS7*	G>A	2,022	1.25 (1.04–1.49)	0.219	0.018	1.27 (1.05–1.54)	0.239	0.015
3	22	rs6001482	*TOP3B, VPREB1*	A>G	2,074	1.25 (1.09–1.44)	0.223	0.002	1.24 (1.07–1.44)	0.214	0.005
4	7	rs12539814	*NOBOX*	A>G	2,075	0.84 (0.72–0.97)	-0.177	0.019	0.83 (0.71–0.98)	-0.181	0.024
5	3	rs4857055	*EPHA6*	C>T	2,065	1.17 (1.02–1.35)	0.160	0.027	1.20 (1.03–1.39)	0.179	0.020
**6**	**1**	**rs117559502**[c]	***SRRM1, CLIC4***	**T>C**	**2,073**	**1.58 (1.08–2.29)**	**0.455**	**0.017**	**1.80 (1.20–2.69)**	**0.587**	**0.004**
7	21	rs116861740[c]	*LINC00478, C21orf37*	G>C	2,070	1.38 (0.95–1.99)	0.320	0.088	1.43 (0.97–2.13)	0.36	0.074
8	12	rs142983199[c]	*AMN1*	C>T	2,072	1.55 (0.97–2.47)	0.435	0.068	1.49 (0.90–2.46)	0.397	0.121
**9**	**18**	**rs62099117**[c]	***SERPINB7, SERPINB2***	**A>T**	**2,055**	**1.70 (1.17–2.48)**	**0.531**	**0.006**	**1.75 (1.16–2.64)**	**0.561**	**0.007**
		GRS3 (Total)	-	-	2,049	2.69 (1.77–4.09)	0.991	<0.001	3.20 (2.03–5.05)	1.164	<0.001
**Males (*n* = 849)**											
1	5	rs13175330	*PAM*	A>G	847	1.28 (0.89–1.84)	0.246	0.183	1.48 (1.00–2.17)	0.389	0.047
2	1	rs1915872	*RGS7*	G>A	829	1.26 (0.97–1.64)	0.229	0.088	1.25 (0.95–1.64)	0.222	0.113
3	22	rs6001482	*TOP3B, VPREB1*	A>G	849	1.19 (0.97–1.46)	0.177	0.087	1.18 (0.95–1.45)	0.162	0.135
4	7	rs12539814	*NOBOX*	A>G	849	0.89 (0.72–1.09)	-0.119	0.263	0.85 (0.68–1.06)	-0.162	0.146
5	3	rs4857055	*EPHA6*	C>T	843	1.10 (0.89–1.35)	0.092	0.389	1.10 (0.88–1.37)	0.095	0.394
6	1	rs117559502[c]	*SRRM1, CLIC4*	T>C	849	1.56 (0.88–2.76)	0.444	0.127	1.67 (0.92–3.05)	0.515	0.092
7	21	rs116861740[c]	*LINC00478, C21orf37*	G>C	848	1.17 (0.69–1.96)	0.153	0.566	1.29 (0.74–2.23)	0.251	0.369
8	12	rs142983199[c]	*AMN1*	C>T	848	1.33 (0.67–2.64)	0.284	0.417	1.40 (0.68–2.88)	0.337	0.359
9	18	rs62099117[c]	*SERPINB7, SERPINB2*	A>T	842	0.96 (0.52–1.78)	-0.038	0.904	1.16 (0.61–2.19)	0.146	0.655
**Females (*n* = 1,226)**											
**1**	**5**	**rs13175330**	***PAM***	**A>G**	**1,222**	**1.63 (1.18–2.27)**	**0.491**	**0.003**	**1.72 (1.20–2.48)**	**0.542**	**0.004**
2	1	rs1915872	*RGS7*	G>A	1,193	1.25 (0.97–1.61)	0.224	0.085	1.31 (0.99–1.73)	0.270	0.057
**3**	**22**	**rs6001482**	***TOP3B, VPREB1***	**A>G**	**1,225**	**1.29 (1.06–1.57)**	**0.252**	**0.012**	**1.29 (1.04–1.61)**	**0.257**	**0.020**
4	7	rs12539814	*NOBOX*	A>G	1,226	0.79 (0.64–0.98)	-0.232	0.031	0.82 (0.65–1.04)	-0.195	0.098

5	3	rs4857055	*EPHA6*	C>T	1,222	1.25 (1.03–1.52)	0.223	0.026	1.30 (1.05–1.61)	0.260	0.017
6	1	rs117559502[c]	*SRRM1, CLIC4*	T>C	1,224	1.64 (0.99–2.73)	0.496	0.055	2.09 (1.20–3.65)	0.737	0.010
7	21	rs116861740[c]	*LINC00478, C21orf37*	G>C	1,222	1.53 (0.91–2.58)	0.425	0.112	1.47 (0.82–2.61)	0.382	0.195
8	12	rs142983199[c]	*AMN1*	C>T	1,224	1.74 (0.91–3.31)	0.552	0.093	1.41 (0.68–2.90)	0.342	0.355
**9**	**18**	**rs62099117[c]**	***SERPINB7, SERPINB2***	**A>T**	**1,213**	**2.63 (1.63–4.25)**	**0.967**	**<0.001**	**2.40 (1.38–4.16)**	**0.874**	**0.002**
		GRS3 (Females)	-	-	1,209	2.79 (1.93–4.04)	1.027	<0.001	2.80 (1.85–4.25)	1.031	<0.001

P values were derived from a logistic regression analysis. [a]Information in the original report is shown. [b]Adjusted for age and BMI. [c]For low-frequency variants with sparse risk-allele homozygotes, a carrier (dominant) coding scheme (0/1; non-carrier vs. carrier of ≥1 risk allele) was used to improve estimate stability and interpretability. Specifically, rs117559502, rs116861740, rs142983199, and rs62099117 were coded as carrier (0/1), whereas all other SNPs were coded additively (0/1/2 risk-allele dosage). "Valid *n*" indicates the number of participants with non-missing genotype data for the corresponding SNP. Weighted GRSs were constructed in the overall group and in females using prespecified selection rules: SNPs were retained for score construction if they (1) showed a statistically significant association with hypertension ($P < 0.05$) in the corresponding group and (2) demonstrated directionally consistent effects under genotype-model checks with stable effect estimates. Risk alleles were aligned so that the retained effects corresponded to a risk-increasing direction for score interpretability. The weighted GRS was computed as the sum of risk-allele scores (coded as 0/1/2 for additive models or 0/1 for carrier models for the SNPs marked with superscript c) multiplied by the corresponding β coefficients obtained from the unadjusted single-SNP logistic regression models fitted under the same coding scheme used for that SNP in the GRS.

Chr, Chromosome; CI, Confidence interval; GRS, Genetic risk score; No., Number; OR, Odds ratio; SNP, Single-nucleotide polymorphism.

Table S6. Stepwise logistic regression for candidate non-genetic predictor selection of hypertension in the total sample

Model	Predictor	B	SE	Wald χ^2	*P*	OR (95% CI)
**Model 1**	ba-PWV (scaled; 1 unit = 100 cm/s)	0.563	0.061	85.968	<0.001	1.755 (1.559–1.977)
**Model 2**	ba-PWV (scaled; 1 unit = 100 cm/s)	0.530	0.061	76.070	<0.001	1.700 (1.509–1.915)
	BMI (kg/m²)	0.190	0.041	21.637	<0.001	1.209 (1.116–1.309)
**Model 3**	ba-PWV (scaled; 1 unit = 100 cm/s)	0.535	0.062	74.941	<0.001	1.708 (1.513–1.928)
	BMI (kg/m²)	0.194	0.041	22.442	<0.001	1.214 (1.120–1.316)
	8-epi-$PGF_{2\alpha}$ (ln-transformed)	0.835	0.267	9.754	0.002	2.305 (1.365–3.894)

Stepwise logistic regression (forward likelihood-ratio method; entry probability = 0.05, removal probability = 0.10) was performed in the total sample using complete cases for hypertension status, ba-PWV, BMI, and 8-epi-$PGF_{2\alpha}$ (N = 786; normal blood pressure *n* = 671, hypertension *n* = 115). ba-PWV was scaled by 100 cm/s (1 unit = 100 cm/s), and 8-epi-$PGF_{2\alpha}$ was ln-transformed. Only predictors retained at each step are shown. The selected non-genetic predictors (BMI, ba-PWV, and 8-epi-$PGF_{2\alpha}$) were applied consistently to the subsequent discrimination (ROC) models to facilitate comparability across groups.

8-epi-$PGF_{2\alpha}$, 8-epi-prostaglandin $F_{2\alpha}$; ba-PWV, Brachial–ankle pulse wave velocity; BMI, Body mass index; CI, Confidence interval; SE, Standard error; OR, Odds ratio.

Table S7. Bootstrap-estimated optimism and optimism-corrected performance metrics (1,000 resamples)

Group	N	Estimated optimism (ΔAUC)	Optimism-corrected AUC (95% CI)	Calibration intercept (95%CI)	Calibration slope (95%CI)	Brier score (95%CI)
Total	775	0.0063	0.827 (0.792–0.867)	-0.002 (-0.232–0.232)	0.965 (0.800–1.161)	0.100 (0.086–0.114)
Males	382	0.0099	0.748 (0.688–0.805)	0.008 (-0.251–0.282)	0.958 (0.713–1.267)	0.142 (0.122–0.164)
Females	397	0.0094	0.904 (0.865–0.948)	-0.007 (-0.488–0.470)	0.927 (0.690–1.221)	0.058 (0.041–0.075)

The ba-PWV variable used here is identical to that used in the regression and ROC analyses presented in Tables 2 and 3. Bootstrap internal validation (B = 1,000) was performed for the final discrimination model defined for each group in Table 3. Specifically, the final model was Model 3 (BMI + ba-PWV + 8-epi-PGF$_{2\alpha}$ + GRS3) in the total sample and in females; GRS3 was constructed separately for the total sample and for females using the corresponding SNP sets and weights defined in Table S5. In males, the final model was Model 2 (BMI + ba-PWV + 8-epi-PGF$_{2\alpha}$), as no GRS was carried forward in the current analysis. Apparent AUCs are shown in Table 3; the present table reports the bootstrap-estimated optimism (ΔAUC) and the resulting optimism-corrected AUCs (apparent AUC − ΔAUC). For calibration, optimism-corrected calibration-in-the-large (intercept) and calibration slope are reported (ideal values: 0 and 1, respectively), together with optimism-corrected Brier scores as a measure of overall performance; 95% CIs were obtained from the bootstrap distribution of the corrected metrics. N denotes the complete-case sample size used for each group-specific model.

8-epi-PGF$_{2\alpha}$, 8-epi-prostaglandin F$_{2\alpha}$; AUC, Area under the receiver operating characteristic curve; ba-PWV, Brachial–ankle pulse wave velocity; BMI, Body mass index; CI, Confidence interval; GRS, Genetic risk score.

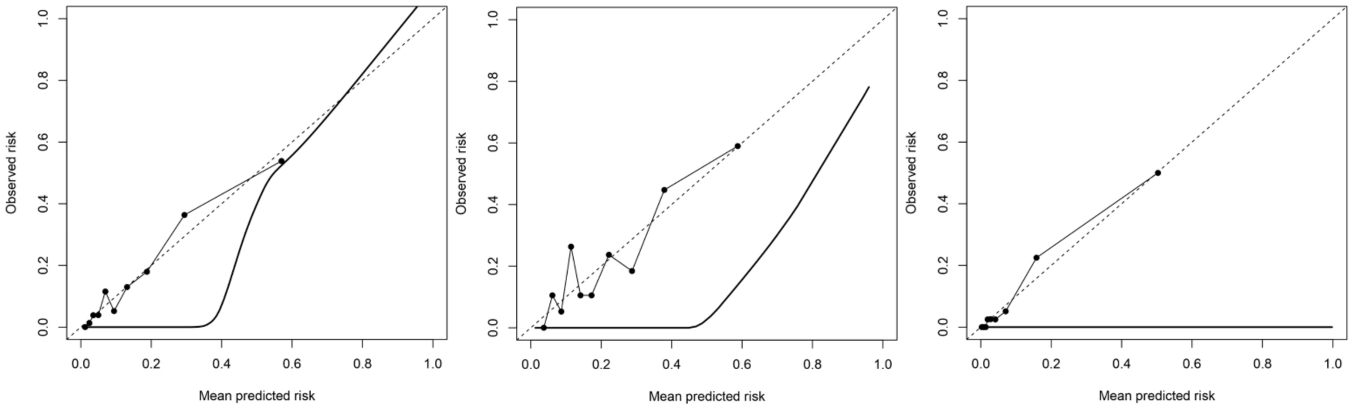

(A) (B) (C)

Figure S1. Calibration plots for the final discrimination models in (A) total, (B) males, and (C) females.

The dashed 45° line indicates perfect calibration. Points represent observed event rates within deciles of predicted risk (g = 10), plotted against mean predicted probabilities in each decile; the solid curve shows a smoothed calibration trend.
